# Lapatinib antitumor effect is associated with PI3K and MAPK pathway: An analysis in human and canine prostate cancer cells

**DOI:** 10.1371/journal.pone.0297043

**Published:** 2024-04-02

**Authors:** Carlos Eduardo Fonseca-Alves, Antonio Fernando Leis-Filho, Zara Alves Lacerda, Patricia de Faria Lainetti, Renee Laufer Amorim, Silvia Regina Rogatto

**Affiliations:** 1 Department of Veterinary Surgery and Animal Reproduction, São Paulo State University–UNESP, Botucatu-SP, Brazil; 2 Institute of Health Sciences, Paulista University–UNIP, Bauru-SP, 17048–290, Brazil; 3 Department of Veterinary Clinic, São Paulo State University–UNESP, Botucatu-SP, 18618–681, Brazil; 4 Department of Clinical Genetics, University Hospital of Southern Denmark, Vejle, Denmark; 5 Institute of Regional Health Research, University of Southern Denmark, Odense, Denmark; Duke University School of Medicine, UNITED STATES

## Abstract

The aberrant activation of HER2 has a pivotal role in bone metastasis implantation and progression in several tumor types, including prostate cancer (PC). Trastuzumab and other anti-HER2 therapies, such as lapatinib, have been used in human breast cancer HER2 positive. Although HER2 overexpression has been reported in PC, anti-HER2 therapy response has revealed conflicting results. We investigated the potential of lapatinib in inhibiting cell migration and inducing apoptosis in two human (LNCaP and PC3) and two canine PC cell lines (PC1 and PC2). Cell migration and apoptosis were evaluated by Annexin V/PI analysis after lapatinib treatment. The transcriptome analysis of all cell lines before and after treatment with lapatinib was also performed. We found increased apoptosis and migration inhibition in LNCaP cells (androgen-sensitive cell line), while PC1, PC2, and PC3 cells showed no alterations after the treatment. The transcriptome analysis of LNCaP and PC3 cell lines showed 158 dysregulated transcripts in common, while PC1 and PC2 cell lines presented 82. At the doses of lapatinib used, we observed transcriptional modifications in all cell lines. PI3K/AKT/mTOR pathway were enriched in human PC cells, while canine PC cells showed enrichment of tyrosine kinase antitumor response and HER2-related pathways. In canine PC cells, the apoptosis failed after lapatinib treatment, possibly due to the downregulation of MAPK genes. Prostate cancer cells insensitive to androgens may be resistant to lapatinib through PI3K gene dysregulation. The association of lapatinib with PI3K inhibitors may provide a more effective antitumor response and clinical benefits to PC patients.

## Introduction

Prostate cancer (PC) is the second leading cause of cancer-related deaths [1] and the initial stages of PC development are driven by testosterone levels [2,3]. Due to the high expression of AR by neoplastic cells and the sensitivity to testosterone levels, chemical or surgical castration is the first standardized treatment for human PC [4,5]. Even achieving good results with castration as a first-line treatment, at later stages, the tumor cells become independent of the AR signaling (hormone-refractory) [4,5]. Consequently, most patients develop resistance to treatment, and the disease progresses [6,7].

Among the several prostate cancer models with biological similarities with human disease, dogs are the only mammal, other than humans, with a high incidence of PC [8]. Preneoplastic lesions and bone metastasis are common findings in human and canine PC [9]. However, a significant difference between dogs and human PC is the dependence on the AR signaling pathway [10]. Even in intact dogs, the canine PC seems to develop from an AR-independent cell and, therefore, is insensitive to androgen hormones [8,10]. Canine PC lacks AR expression and has no dependency on testosterone levels, and castration confers no clinical benefits [8,10].

The LNCaP and PC3 cells express EGFR and ERBB2, and dysregulation of both receptors seems to play a major role in PC progression. Androgen hormones regulate prostate function in a complex mechanism involving testosterone metabolism [11]. This activation mechanism downregulates several growth pathways, including the epidermal growth factor receptor (EGFR) pathway at a transcriptional level [12]. The EGFR family is a tyrosine kinase group of proteins composed of four types of transmembrane receptors, in which EGFR/ERBB1 and ERBB2/HER2/NEU are the most studied [13,14]. EGFR and ERBB2 are activated by different mechanisms, including dimerization [13] and phosphorylation [15], and their role in PC is still controversial. In canine PC the role of ERBB2/EGFR1 has also been explored. Sakai et al. [16] reported ERBB2 copy number gains and increased gene expression in eight of 13 canine PC samples. High ex-pression levels of ERBB2 have been described in canine PC [17]. In a larger study investigating 25 canine PC, eight cases showed high EGFR expression, whereas 21 samples overexpressed ERBB2 [18].

Lapatinib is a potent and selective dual TKI that blocks EGFR and ERBB2 targets [19,20]. Lapatinib has been tested in different clinical trials of human PC [21–24]. A phase II clinical trial enrolling castration-resistant PC patients (N = 29) treated with lapatinib verified an antitumor activity in a small subset of cases [25]. The authors hypothesized that the lack of clinical efficacy was associated with the study design without time-to-event endpoints [25]. On the other hand, a multicenter phase II clinical trial in advanced untreated hormonally PC patients (N = 23) showed that lapatinib was well tolerated, but no significant antitumor activity was found in PC patients at the early stages of the disease [24]. A plausible explanation for these controversial results is the absence of homogenous inclusion criteria and the small set of enrolled patients in these studies. The effectiveness of lapatinib in prostate cancer treatment and the mechanisms involved in the therapy response are still unclear [21].

The characterization of dogs as a model for human PC has provided valuable information regarding tumor biology and antitumor response [26,27]. The pharmacologic mechanisms and antitumor response of lapatinib could be explored and understood better using dogs as cancer models [28,29]. Dogs with advanced stage bladder cancer were treated with lapatinib as first-line treatment, showing a high response rate [30]. The authors concluded that lapatinib could be introduced as a first-line treatment in dogs with advanced bladder cancer. These results encouraged the use of lapatinib for human bladder cancer and demonstrated the importance of dogs in future studies with this drug. Comparative studies of PC in humans and canines can potentially elucidate the antitumor effect of lapatinib in these tumors.

Since no previous study compared the lapatinib antitumor effect in human and canine PC cells and that lapatinib could be promising for therapy in both species, we performed transcriptome of human and canine PC cell lines treated with lapatinib aiming to identify the functional network related to the drug response.

## Results

### Lapatinib IC_50_ of cancer cell lines

The half maximum inhibitory concentration (IC50) was assessed to measure the lapatinib efficacy in all four cell lines using MTT assay((3-(4,5-Dimethylthiazol-2-yl)-2,5-Diphenyltetrazolium Bromide). The LNCaP and PC3 human cell lines presented IC50 of 0.48 μM and 0.36 μM, respectively, while the canine PC1 and PC2 cell lines showed an IC50 of 0.22 μM and 0.26 μM, respectively (S1 Fig).

### Apoptosis and migration assays

**Fig 1:**
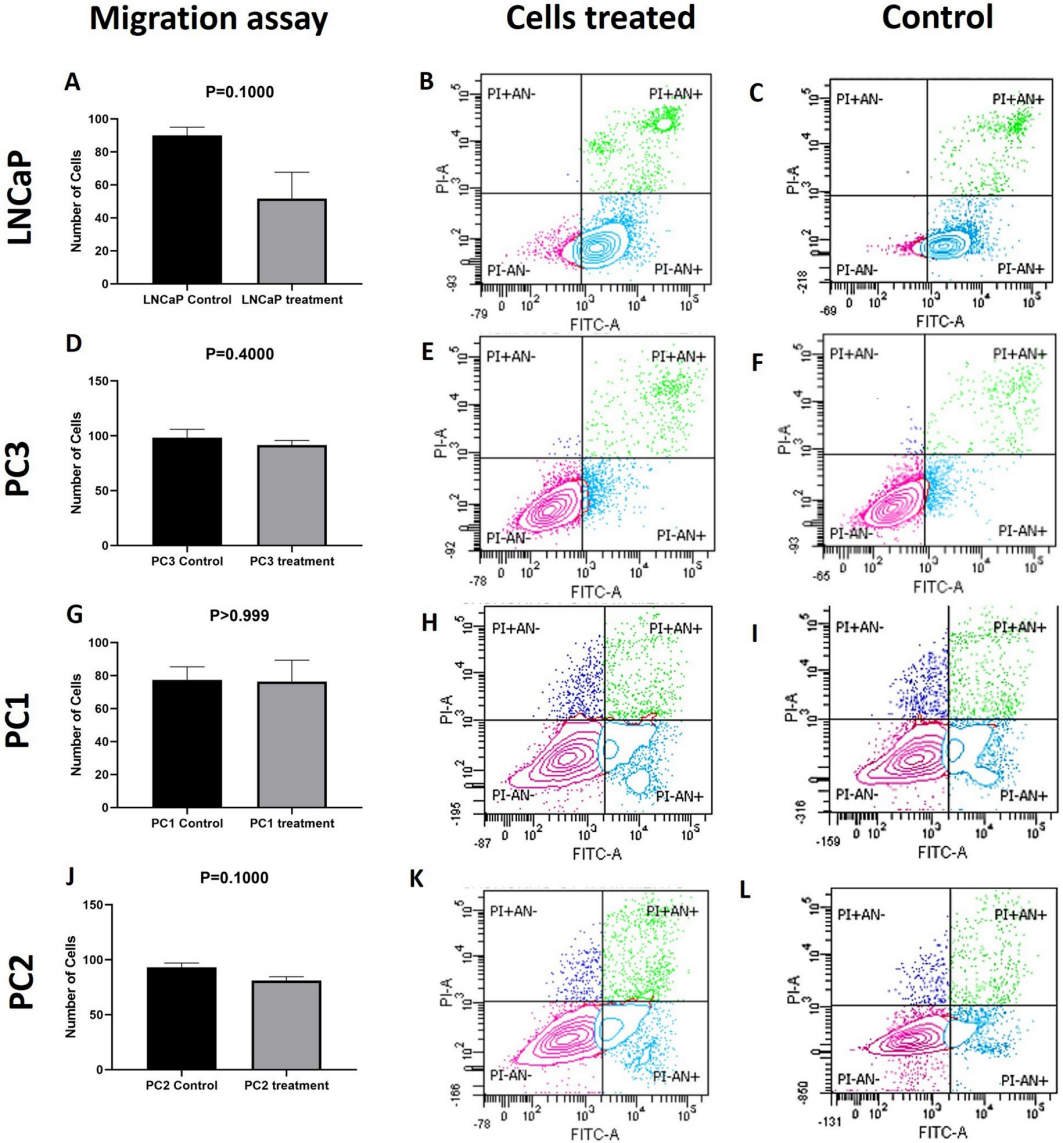
Apoptosis and migration assays performed in human (LNCaP and PC3) and canine (PC1 and PC2) cell lines. No significant difference was observed in the migration of LNCaP (A), PC3 (D), PC1 (G), and PC2 (J) cells. LNCaP cells treated with lapatinib showed a significant difference in apoptosis and necrosis (B) while no difference was observed in PC3 (E), PC1 (H), and PC2 (K) cell lines. Cell lines treated with DMSO (control) presented viability of 87.9% for LNCaP (C), 90.4% for PC3 (F), 76.1% for the canine PC1, and 83% for the canine PC2. PI: Propidium iodide; AN: Annexin; (+): Positive, (-): Negative; PI-/AN-: Viable cells (alive); PI-/AN+: Early apoptosis; PI+/AN+: Late apoptosis; PI+/AN-: Necrotic cells.

**Fig 2:**
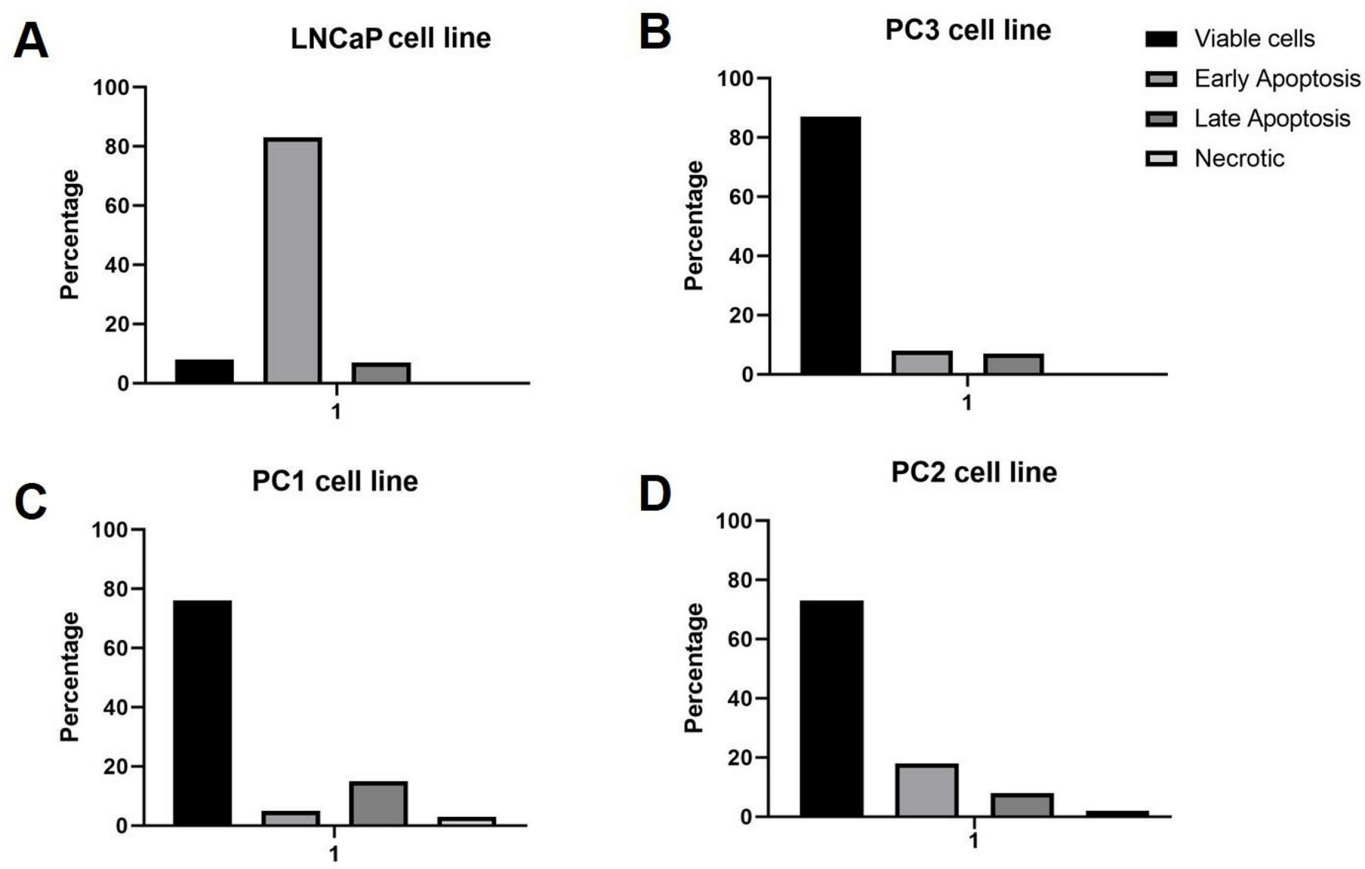
Graphic representation of the apoptotic assay. A: The LNCaP cell line presented more than 80% of early apoptosis. The human PC3 (B), canine PC1 (C), and PC2 (D) cell lines showed over 70% of viable cells and a low apoptotic rate.

Since MTT is used to assess cell viability as a function of redox potential, i.e., reduction of cellular activity, its results are not necessarily correlated with apoptosis and necrosis at the IC_50_ dosage [31]. Therefore, apoptosis and necrosis rates were measured using the lapatinib IC_50_ for each cell line. No significant differences in apoptosis or necrosis were observed comparing treated and non-treated PC3 cells (Figs 1 and 2). However, the LNCaP cell line showed 83.6% of apoptosis and 0% of necrosis after the same treatment conditions (Fig 2). In canine PC1 and PC2 cell lines, no significant difference was detected in the number of cells in apoptosis or necrosis after 24 hours of treatment. Although the migration of untreated LNCaP cells was higher than treated cells, no statistical significance was found. The treatment with lapatinib showed no alterations in cell migration in the other cell lines (Fig 1).

### Transcriptomic profile

We treated human and canine cells with lapatinib IC_50_ to evaluate the dysregulated genes in response to the treatment. The transcriptional analysis was performed by comparing treated and non-treated human and canine cell lines. The LNCaP cell line presented 6073 dysregulated transcripts (Fig 2), including 3493 upregulated and 2580 downregulated genes (S1 Table). Among the differentially expressed genes, we identified the upregulation of *PIK3R1*, *PIK3CB*, *PTK2*, and *TARF3* and the downregulation of *EGFR*, *FGF2*, and *PDK1* genes. The PC3 cell line showed 2905 dysregulated transcripts (1384 up- and 1521 downregulated), including the upregulation of *STEAP4*, *RELB*, and *MAP3K14* and downregulation of *CYP1B1*, *PTEN*, *PDK1*, and *ACOT2*. The LNCaP and PC3 cell lines showed 158 commonly dysregulated transcripts, including *DBNDD2* and *TTC1* upregulation and *SCG5*, *ARMC2*, *SNORD63*, *SNORD77*, *TMSB4Y*, and *APCDD1L-AS1* downregulation.

**Fig 3:**
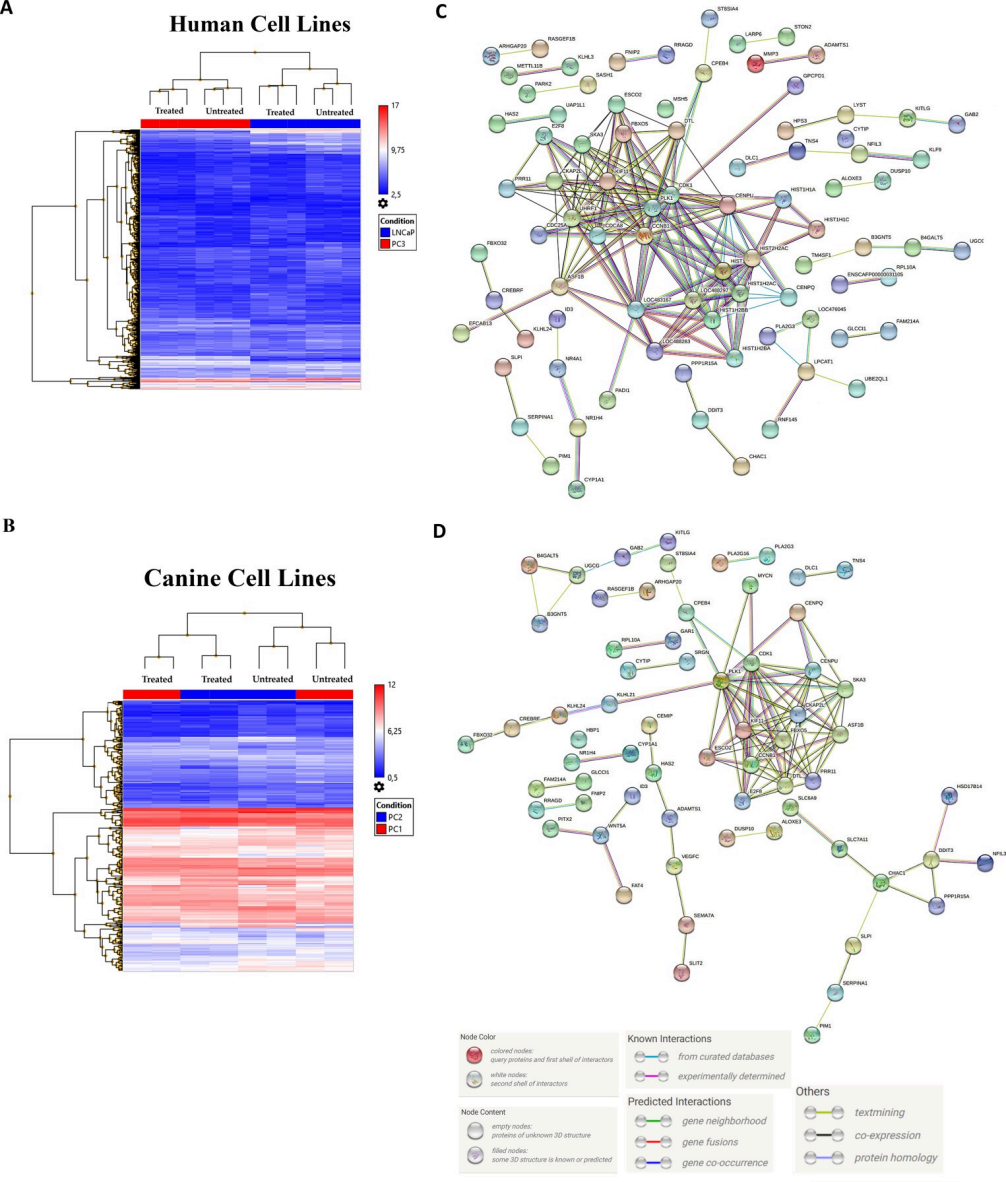
Transcriptomic analysis comparing human (A) and canine (B) prostate cancer cells treated and non-treated with lapatinib. Hierarchical clustering analysis of the differentially expressed genes between treated and non-treated cell lines with lapatinib. Red and blue colors indicate up or downregulated genes, respectively. The mean signals were corrected and transformed to the log2 scale, and genes with at least 1.5-fold changes with p < 0.05 at the 95% confidence level were considered significant. C: Protein-to-protein integration network of LNCaP and PC3 cells treated and non-treated with lapatinib. Several complex interactions among proteins were observed, with CDK1, PLK1, and CCNB1 proteins in the middle of the interaction map with the highest number of interactions. D: Protein-to-protein integration network of canine PC1 and PC2 cells treated and non-treated with lapatinib showing fewer interactions than those observed in human prostate cancer cells. However, the proteins with the highest number of interactions are similar in human and canine cells (PDK1 and PLK1).

The PC1 canine cell line presented 613 differentially expressed genes (DEGs), including 224 upregulated and 389 downregulated. Among them, *BRIX1*, *FLRT3*, *PTK2* and *CYP27A1*, *ALCAM*, and *RRAGD* were downregulated. The complete list of dysregulated genes is detailed in S2 Table. The PC2 cell line presented 570 DEGs, with 203 upregulated and 367 downregulated genes. The *WNT5A*, *CD1A6*, and *PLA2G3* genes were upregulated and *MMP3*, *MMP1*, and *FGFR2* were downregulated. The canine PC1 and PC2 cell lines showed 82 commonly dysregulated transcripts, including *E2F8*, *PARK2*, *GAB2*, and *AQP9* genes. A representative heatmap of the differentially expressed genes comparing treated and non-treated cell lines with lapatinib is shown in Fig 3.

### Pathways associated with the lapatinib treatment

Gene set enrichment analysis was performed to identify pathways potentially dysregulated in cells treated with lapatinib. The human LNCaP cell line presented an enrichment of genes involved in the PI3K-AKT-mTOR signaling pathway, EGFR TKI resistance, ErbB signaling pathway, and focal adhesion. Several signaling pathways were found in the PC3 cell line, including PI3K-AKT-mTOR, TNF-related weak inducer of apoptosis (TWEAK), DNA damage response (only ATM dependent) and senescence, and autophagy in cancer. The complete list of pathways is described in S3 Table.

The canine PC cells presented an enrichment of tyrosine kinase antitumor response and HER2-related pathways, among others. The PC1 cell line presented the MAPK, EGFR1, and androgen receptor signaling pathways. Several pathways related to the MAPK signaling pathway were found in the canine PC2 cell line (S4 Table).

### Protein-protein interaction (PPI) and Gene Ontology

**Fig 4:**
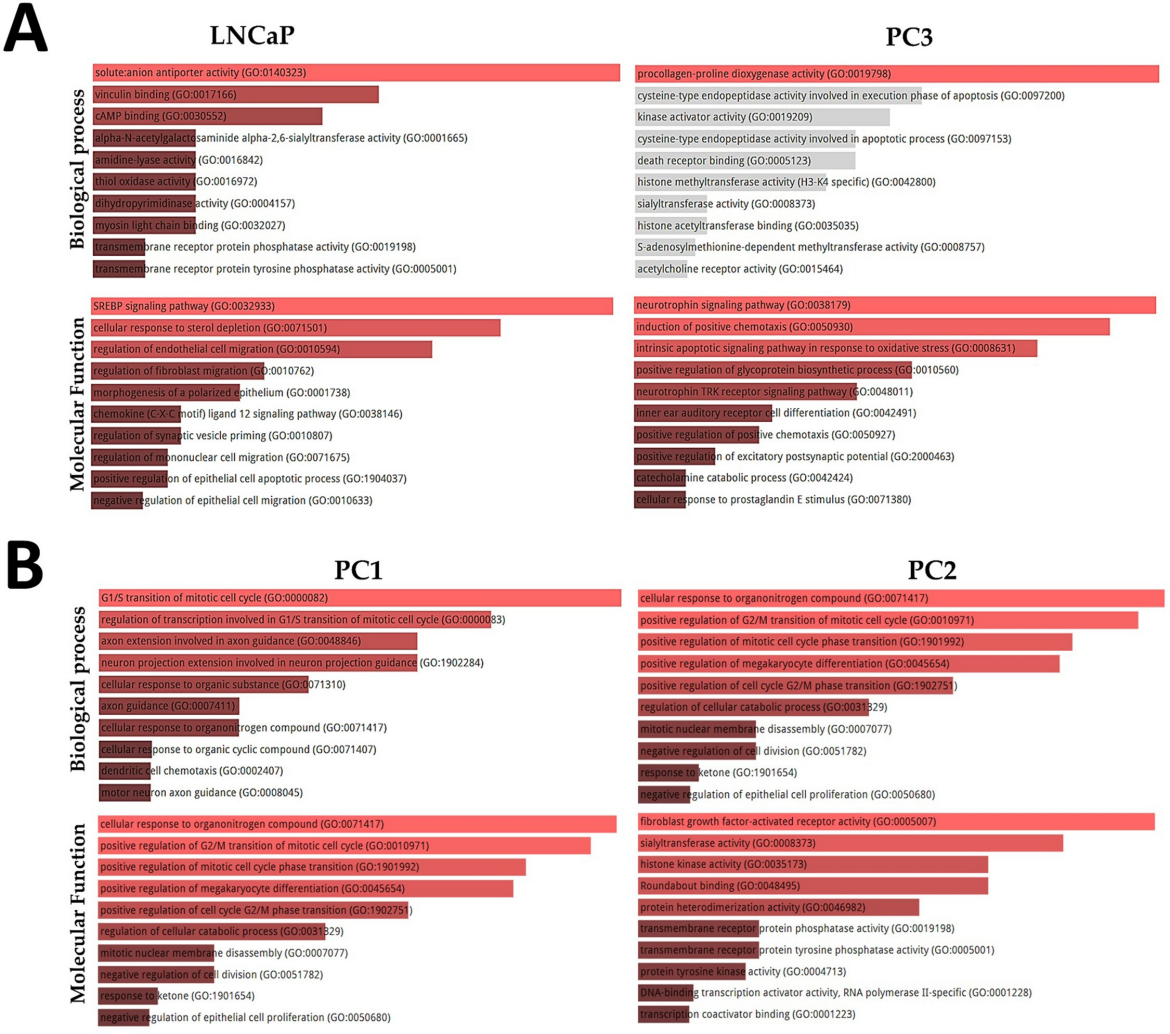
A: TOP 10 biological and molecular ontology processes, respectively, of the LNCaP and PC3 cell lines. Different terms were found for each cell line. LNCaP cell line showed terms associated with modification of epithelial cell morphogenesis, including cell migration. PC3 cell line presented processes related to oxidative stress and positive regulation of chemotaxis. B: TOP 10 biological and molecular ontology processes for canine PC1 and PC2 cell lines. PC1 cells presented several processes related to cell division and cell cycle alterations. PC3 cell line showed different processes related to cell binding and heterodimerization, which were related to EGFR tyrosine kinase inhibitor regulation.

The PPI analysis revealed that EGFR, PI3KR1, and H4C1 proteins presented a higher number of interactions in the human LNCaP cell line (Fig 3). The PC3 cell line showed fewer interactions among proteins than LNCaP cells, in which GOLGA2 and MAP3K14 proteins presented higher interactions (Fig 3). Gene ontology analysis showed that differentially expressed genes were significantly enriched in Biological and Molecular processes (Fig 4A). Among the ontology processes, the transmembrane receptor protein phosphatase activity, negative regulation of epithelial cell migration, and morphogenesis of a polarized epithelium were related to the lapatinib antitumor effect. The complete list of ontologies can be found in S5 Table.

Both canine PC cell lines showed a complex protein interaction network. PCNA, LRRK2, and PRKN presented a higher number of complex interactions in the PC1 cells, while a similar pattern was observed involving PLK1, AURKB, PRKN, and CCNB1 in PC2 cells (Fig 3). The ontology analysis revealed several processes related to tyrosine kinase, including Protein Tyrosine Kinase Activity, Protein Heterodimerization Activity, and Signaling Receptor Complex Adaptor Activity (Fig 4B). The complete list of ontology terms is detailed in S6 Table.

## Discussion

Human prostate cancer arises from a luminal androgen receptor-positive cell and is sensitive to testosterone levels. At the early stages of the disease, the patients are surgically or chemically castrated due to the high expression of AR by neoplastic cells and the sensitivity to testosterone levels [2]. However, the disease may evolve into a drug-resistant form due to failure after androgen deprivation therapy [32]. Aberrant activation of HER2 is involved in bone metastasis implantation and cancer progression, which has been the rationale for designing clinical trials testing HER2 inhibitors in PC patients [25]. Lapatinib is an anti-HER2 therapy used as an alternative to trastuzumab in breast cancer [19] and an option to treat hormone-refractory prostate cancer patients.

A subset of human PC patients will develop an aggressive variant with a high metastatic rate and poor antitumor response, named castration-resistant prostate cancer [31]. Tumors showing neuroendocrine differentiation may evolve to an androgen-negative prostate cancer [31]. Interestingly, prostate cancer in dogs is androgen insensitive and negative to androgen receptor [8]. Therefore, dogs are an attractive model for drug assays since they have similar physiological parameters (including the immune system) to humans, among other features [32]. Based on previously published data [23–25], we hypothesized that human and canine PC cell lines insensitive to androgen hormones would present a most effective antitumor response to lapatinib and higher apoptosis compared to human-sensitive cell line LNCaP. However, the opposite result was found. Lapatinib has been proposed as an alternative to treat urological cancers in dogs at clinical routine [30]. Our study focused on the lapatinib antitumor effect independently of other chemotherapeutic agents that are not routinely used in the clinical practice of canine PC.

The LNCaP cell line showed the lowest lapatinib IC_50_ and a higher apoptosis rate than the human PC3 cell line and both canine cells, indicating a most effective antitumor response in vitro. According to these results, we performed a transcriptome analysis and identified a higher number of dysregulated genes in the LNCaP cell line compared to the other three cell lines. Interestingly, the LNCaP cell line presented diverse genes (such as upregulation of *PTEN*, *PIK3CB*, and *PIK3R1* and downregulation of *FOXO1* and *PDK1)* from the PI3K-AKT-mTOR signaling pathway dysregulated. PI3K/AKT signaling is an oncogenic pathway responsible for cell migration, proliferation, and drug resistance in several tumor types. The PI3K/AKT pathway activation is a major downstream consequence of ErbB gene phosphorylation; both events are responsible for alternative activation of the AR pathway and linked to hormone stimulation [33–35]. The upregulation of PI3K pathway genes may be associated with *AR* stability during this gene phosphorylation, activating an AKT-independent mechanism to induce cell proliferation [36]. Also, the PI3K signaling pathway can activate RAS, AKT, MAPK, and interplay with AR signaling pathway. Therefore, PI3K signaling pathway activation can directly activate several oncogenic pathways in PC [37].

The androgen-insensitive human PC3 cell line also presented dysregulation of the PI3K-Akt signaling pathway. However, a different list of differentially expressed genes was obtained according to the lapatinib treatment. While the LNCaP cell line activated the PI3K pathway related to phosphorylation, the human PC3 cell line activated the pathway related to the TWEAK complex. TWEAK regulates cell morphology by modifying junctional proteins, leading to an epithelial-mesenchymal phenotype [38]. Although both cell lines presented PI3K pathway dysregulation, different genes had altered expression, and consequently, distinct mechanisms of response to lapatinib treatment were activated. The PC3 cell line presented upregulation of *IGF1R*, *LPAR2*, *GNG7*, *EIF4EBP1* and downregulation of *BDNF* and *JAK2*, *LAMC2*, *PTEN*, *VEGFA*, *PPP2CA*, *IRS1* and *NTF3* after lapatinib treatment. The IGFR1 has been implicated with the AR as an alternative route of activation of the AR pathway, independent of androgen hormones, leading to the castration-resistant phenotype [39]. Therefore, the upregulation of *IGFR1* and related genes could be associated with poor lapatinib response and apoptosis resistance.

In castration resistant prostate cancer (CRPC), it is widely accepted that PI3K/AKT/mTOR pathway plays a major role in intracellular phosphorylation, inducing cellular proliferation, survival, and angiogenesis [40]. Therefore, the combination of lapatinib and inhibitors of specific PI3K/AKT/mTOR pathway components might present a better antitumor response in CRPC.

It is important to highlight that LNCaP is an androgen-sensitive cell line, and PC3 is an androgen-resistant cell line. LNCaP cells express AR in the nucleus, and the PC3 cell line shows a lack of AR nuclear expression [41,42]. Therefore, it is plausible to suggest that both cell lines activated the PI3K pathway through the AR signaling pathway, even in cells with low AR expression. On the other hand, both canine cell lines are insensitive to androgen hormones and AR-negative [43], which could explain why canine cell lines did not deregulate the PI3K pathway and bring an interesting perspective on lapatinib treatment. Our results suggest that activation of the AR signaling pathway leading to the reversion of androgen insensitive status may enhance lapatinib antitumor response.

The canine PC cells presented resistance to lapatinib treatment, with a high IC_50_ (dosage incompatible with an in vivo translation), and apoptosis failed after treating both cells with the IC_50_ dosage. Apoptosis may not have been identified for different reasons. First, MTT is a metabolic assay and not a specific assay for viability evaluation [44]. Therefore, cells could only be presenting decreased metabolic activity instead of being induced to death. Also, annexin and propidium iodide assays evaluate apoptosis and necrosis but no other forms of cell death. Multiple mechanisms have been related to cell death in addition to apoptosis and necrosis, including programmed non-apoptotic cell death (PNACD). PNACD encompasses various mechanisms and phenotypes, such as cell death with vacuole involvement, cell death dependent on mitochondria, cell death influenced by iron levels (ferroptosis), and cell death associated with immune responses [45].

The transcriptome of both canine cell lines after lapatinib treatment revealed a low number of dysregulated genes after treatment. Evaluating the pathways associated with lapatinib treatment, we found that most dysregulated genes were related to modifications in cellular structure, including cell morphology. However, both cells stimulated androgen receptor pathways through *BMF* downregulation. The suppression of *BMF* gene expression was associated with preventing cell apoptosis induced by androgen ablation and promoting hormone-independent growth [42]. Thus, even presenting AR negative expression, canine cells can have stimulated an AR-related pathway to avoid apoptosis [46], which explains the results obtained in PC canine cells.

MAPK pathway genes were suppressed after lapatinib treatment in both canine cell lines. Also, the MAPK pathway is related to the AR signaling pathway and responsible for a non-genomic activation of this pathway [43]. MAPK pathway may activate the AR signaling pathway without AR expression or the presence of androgen hormones that activate AR [47]. The androgen resistance in human prostate cancer is also driven by the MAPK pathway [48]. Our canine prostate cancer cells presented downregulation of the MAPK pathway genes, which are related to phagocytosis and autophagy. Therefore, treating cells with lapatinib induces the downregulation of genes related to cell destruction and apoptosis, which may prevent apoptosis through the MAPK pathway.

## Materials and methods

### Ethics statement

This study was approved by the Ethics Committee on Animal Care at the School of Veterinary Medicine and Animal Science of the São Paulo State University (Protocol: 0004/2017). The patient´s owner written a signed consent approving the use of the dog´s tissue for development of each canine cell line.

### Cell cultures

The canine prostate cancer cell PC1 was established from a fresh tissue from a 10-year-old mixed breed dog with non-metastatic tumor and the PC2 was established from an 11-year-old poodle dog with metastatic tumor, both were previously established and characterized [44] in accordance with the institutional ethics guidelines. PC1 and PC2 cells at passage 20 were cultured in DMEN/F12 medium (Lonza, Basel, Switzerland) supplemented with 10% of fetal bovine serum (Lonza, Basel, Switzerland) and 1% of penicillin and streptomycin (Lonza, Basel, Switzerland). The human cell lines LNCaP (passage 15) and PC3 (passage 23) were retrieved from the European Collection of Authenticated Cell Culture (ECACC) and American Type Culture Collection (ATCC), respectively. These human cell lines were cultured in RPMI medium (Sigma Aldrich, Saint Louis, MO, USA) supplemented with 10% of fetal bovine serum (Lonza, Basel, Switzerland) and 1% of penicillin and streptomycin (Lonza, Basel, Switzerland). All cell lines were negative for mycoplasma contamination. Table 1 summarizes the main characteristics of the cell lines used in our study.

**Table 1:** Main features of human (LNCaP and PC3) and canine (PC1 and PC2) prostate cancer cell lines.

Cell line	Origin	PSA expression	AR expression	Mutational Status	Cell line derived
LNCaP	Human	Positive	Positive	*AR* p.Thr878Ala (c.2632A>G); *MEN1* p.Tyr318Ter (c.954T>G) (p.Tyr313Ter, c.939T>A) *PIK3R1* p.Arg639Ter (c.1915C>T) *PTEN* p.Lys6Argfs*4 (c.17_18delAA)	lymph node metastasis
PC3	Human	Negative	Negative	*TP53* p.Lys139Argfs*31 (c.414delC) (413delC)	bone metastasis
PC1	Canine	*	Negative	Na	non-metastatic carcinoma
PC2	Canine	*	Negative	Na	metastatic carcinoma

PSA: Prostate specific antigen; AR: Androgen receptor; na: Not available; dogs have no *PSA* gene.

### Determination of IC50

The half-maximal inhibitory concentration (IC_50_) for each cell line was determined using the assay based on the cleavage of tetrazolium yellow salt MTT [3- (4,5-dimethylthiazol-2-yl) -2,5-diphenyl tetrazolium bromide]. All cells were seeded in a 96-well plate containing a complete cell culture medium, as described above, for 24 hours at 37°C. Next, the cells were incubated in a medium serum-free and lapatinib at 2μM, 4μM, 6μM, 8μM, 10μM, 12μM, 14μM, and 16μM for 24 hours. For MTT control, we used cells naïve to treatment (basal control) and cells treated with the highest DMSO (dimethyl sulfoxide) concentration (control of DMSO toxicity). In the same plate, each dosage was tested in triplicate, and each replicate in triplicate (3x3). Ten μL of MTT labeling reagent was added to each well, followed by incubation at 37°C for 4h. The spectrophotometric absorbance of the samples was detected using a microtiter plate reader at 570nm.

### Necrosis and apoptosis assays

To evaluate necrosis and apoptosis induced by the treatment with lapatinib IC50, the annexin V/propidium iodide (PI) apoptosis assay (Sigma-Aldrich, Saint Louis, MO, USA) was performed using flow cytometry. Briefly, cell lines were treated with lapatinib IC_50_ dosage for 24 h, harvested, and counted. Subsequently, 1×10^5^ cells were added in 200 μL of annexin V binding buffer containing 4 μL of 0.5 mg/mL PI and 2 μL of annexin V-FITC and incubated for 15 min at room temperature in the dark. Flow cytometry was performed as previously described [49]. Cell lines treated only with DMSO (diluent for lapatinib) were used as negative controls. Cells presenting annexin V and PI negative were classified as viable; cells expressing annexin V and PI negative were considered in early apoptosis; cells positive for both annexin V and PI were classified as late apoptosis, and cells negative for annexin V and positive for PI were considered necrotic [49].

### Transcriptomic analysis

The mRNA extraction was performed in duplicate for canine cells and triplicate for human cells. Cells were seeded in a 6-well plate containing medium supplemented with fetal bovine serum and antibiotics for 24 hours. Based on the IC50 values, cells treated with DMSO (control) and lapatinib were incubated at 37°C. After 24 hours, the medium was discharged, and cells were washed with PBS (phosphate saline buffer) (Sigma Aldrich, Saint Louis, MO, USA) two times. mRNA was extracted using Qiagen® RNeasy Mini Kit (Hilden, Germany), according to the manufacturer’s recommendations. RNA quality and quantity assessments were conducted using an RNA 6000 Nano labchip (Bio-analyzer, Agilent, Santa Clara, CA, USA) and Nanodrop spectrophotometer (Thermo Fisher Scientific, Waltham, MA, USA), respectively.

The transcriptomic profiling of human cell lines was evaluated using the Clariom™ D Assay platform (Thermo Fisher Scientific, Waltham, MA, USA). Labeling, hybridization, and washing followed the manufacturer’s recommendations. Normalization, quality control, and analysis were assessed by the Transcriptome Analysis Console (TAC software Thermo Fisher Scientific, Waltham, MA, USA, v.4.0). The canine transcriptome analysis was performed using the Affymetrix Canine Gene 1.0 ST Array (Affymetrix, Santa Clara, CA, USA). Data were extracted using the Affymetrix Genotyping Console (Affymetrix) and normalized using quantile normalization and robust multi-array analysis (RMA) background correction. Differentially expressed genes (fold-change of 1.5 and p-value < 0.05) were obtained by comparing the cell lines in the same conditions treated or not with lapatinib.

The enrichment of gene ontology (GO) process of differentially expressed genes was performed using Enrichr (https://amp.pharm.mssm.edu/Enrichr/, accessed on 15 November 2022). Protein-protein interactions were visualized and analyzed using PINA v3.0 (https://omics.bjcancer.org/pina/home.action, accessed on 15 November 2022).

## Conclusion

The lapatinib treatment induces alteration in the PI3K pathway in human prostate cancer cells. Prostate cancer cells insensitive to androgens can be resistant to lapatinib through PI3K gene dysregulation. Therefore, the association between lapatinib and PI3K blocker could provide a more effective antitumor response. In canine prostate cancer cells, the apoptosis failed after lapatinib treatment, possibly due to the downregulation of MAPK genes related to apoptosis and autophagy.

## Supporting information

S1 Fig Lapatinib half maximal inhibitory concentration for each cell line. (DOCX)

S1 Table Dysregulated genes in LNCaP and PC2 cell lines. (XLSX)

S2 Table Dysregulated genes in PC1 and PC2 canine cell lines. (XLSX)

S3 Table Pathways associated with LNCaP and PC3 cell lines. (XLSX)

S4 Table Pathways associated with PC1 and PC2 canine cell lines. (XLSX)

S5 Table Ontologies associated with LNCaP and PC3 cell lines. (XLSX)

S6 Table Ontologies associated with PC1 and PC2 canine cell lines. (XLSX)
