# Supplementary material for: Lapatinib antitumor effect is associated with PI3K and MAPK pathway: An analysis in human and canine prostate cancer cells
Source: PLoS One. 2024 Apr 2;19(4):e0297043. doi: 10.1371/journal.pone.0297043 (PMC10986952; doi:10.1371/journal.pone.0297043)
Supplement: S1 Fig — (DOCX) [file pone.0297043.s001.docx]

**
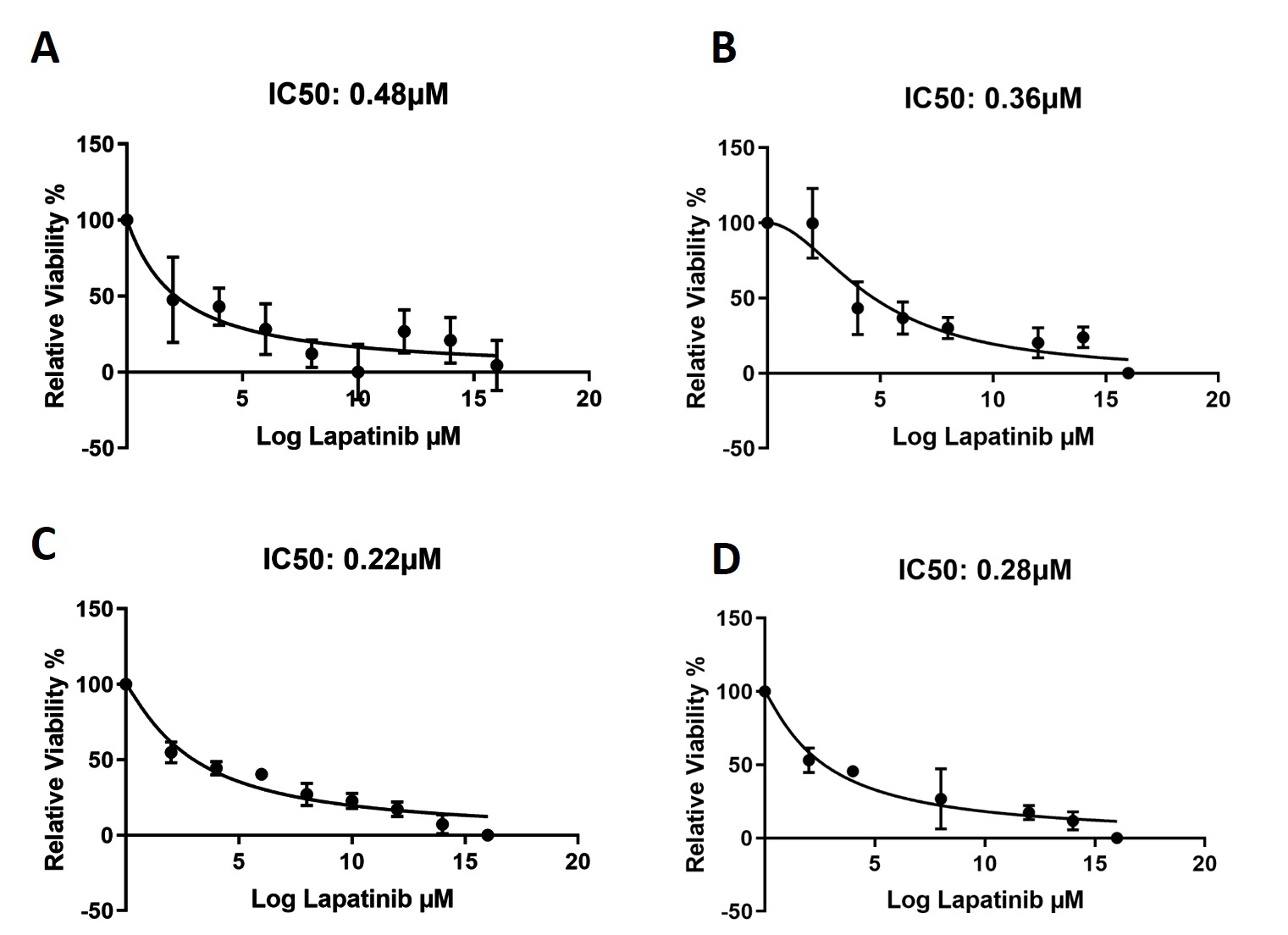
**

**Supplementary Figure 1**. Lapatinib IC_50_ curves in human and canine prostate cancer cell lines. The IC_50_ was quantified using a crystal violet assay following 24 hours of treatment with lapatinib (1-100 µM). The measurements were performed in triplicates. A: LNCaP cell line presented IC_50_= 0.48µM. B: PC3 cell line showed an IC_50_ of 0.36µM. C and D: canine cells lines presented a similar IC_50_ (0.22µM and 0.28 µM, respectively).
